# General and Domain-Specific Contributions to Creative Ideation and Creative Performance

**DOI:** 10.5964/ejop.v12i4.1132

**Published:** 2016-11-18

**Authors:** Donggun An, Mark A. Runco

**Affiliations:** a Center for Learning Science and Creative Talent Development, Seoul National University, Seoul, Korea; b Department of Educational Psychology, University of Georgia, Athens, GA, USA; University College Cork, Cork, Ireland

**Keywords:** general creativity, domain-specific creativity, creative ideation, creative performance

## Abstract

The general objective of this study was to reexamine two views of creativity, one positing that there is a general creative capacity or talent and the other that creativity is domain-specific. These two views were compared by (a) testing correlations among measures of domain-general and domain-specific creativity and (b) examining how the general and the specific measures was each related to indices of knowledge, motivation, and personality. Participants were 147 college students enrolled in a foreign language course. Data were collected on participants’ domain knowledge, motivation, and creative personality, as well as four measures representing “General or Domain-Specific Creative Ideation” or “Creative Performance and Activity”. Results indicated that the four measures of creativity were correlated with one another, except for “General Performance and Activity” and “Domain-Specific Ideation.” A canonical correlation indicated that knowledge, motivation, and personality were significantly correlated with the four creativity measures (Rc = .49, p < .01). Multiple regressions uncovered particular relationships consistent with the view that creativity has both general and domain-specific contributions. Limitations, such as the focus on one domain, and future directions are discussed.

## Introduction

There is a debate whether creativity is domain-general or domain-specific (e.g., Baer, 1998; Plucker, 1998). Plucker (1998) described a tendency to study creativity with an assumption of generality (e.g., Guilford, 1967; Torrance, 1974).The key evidence for this position is that measures of creativity within particular domains (usually creative performance and activity check lists) show significant intercorrelations. Runco (1987), for example, assessed both gifted and non-gifted children’s creative performance and activity and found significant correlations among the indices in various domains (e.g., writing, music, crafts, performing arts, and science). Hocevar’s (1976) study indicated that for college students, significant correlations were found among creative performance and activity in various fields (e.g., fine arts, crafts, performing arts, math-science, and music). These studies support the hypothesis that the nature of creativity is general rather than domain-specific.

The domain-specificity view holds that creativity emerges within fields. The critical evidence for this position is based on low correlations among different domain-derived artifacts created by the same person. For example, Baer (1991) reported that for eighth graders, non-significant correlations were found among diverse domain-specific products (i.e., mathematical, verbal, and combined). Runco (1989) reported similarly low correlations (average *r* = .18) among three different types of artwork in a group of 4th, 5th, and 6th grade students. Baer (1993) found non-significant correlations among different domain-oriented products (e.g., poems, math puzzles, and collages) in a sample of diverse age groups (elementary children to adults). Also relevant are the weak relationships between domain-general creativity and domain-specific creativity. Han (2003) showed that second-graders’ general divergent thinking was not correlated with their domain-specific artifacts in language, art, and mathematics. Diakidoy and Spanoudis (2002) found that ninth-graders’ performances (fluency and originality) differed significantly on general and domain-specific divergent thinking tests. These studies support the hypothesis that creativity is domain-specific.

One important difference between the two views of creativity involves contributions of knowledge and personality. Theories of domain-specific creativity often emphasize knowledge, and in particular domain-specific knowledge (e.g. Vincent, Decker, & Mumford, 2002; Weisberg, 2006). Although academic grades are generally unrelated to creativity (Holland, 1961; Holland & Richards, 1965), domain-specific knowledge influences domain-specific creative performances (Amabile, 1990; An, Song, & Carr, 2016; Vincent et al., 2002). Similarly, domain-specific creativity is associated with personality, different domains having different profiles of contributing traits (e.g., Feist, 1998, 1999).

The primary objective of the present research was to offer a fine-grained examination of how both general and domain-specific creativity are related to knowledge, motivation, and personality. This research is in fact unique in that it included measures of knowledge, motivational, and personality variables and examined each as predictor of both general and domain-specific creativity. An et al. (2016) had previously assessed knowledge, motivation, and personality but they looked to different forms of creativity (divergent thinking and creative expert performance) in only one domain (educational psychology) and did not take into account general creativity. The present study is different from An et al. (2016) in that the present study compared general and domain-specific creativity rather than different forms of creativity in one domain. The present study also measured the quantity of creativity, not just the quality of creativity. Of course the samples in this investigation and that of An et al. (2016) were different as well. An et al. sampled Korean college students. And here samples were entirely comprised of college students from the U. S. The results of the present study were expected to offer a useful perspective on the debate concerning general and domain-specific creativity.

## Method

### Participants

Participants included a total of 147 undergraduates from a large university in the southeastern United States. Each student was enrolled in a Korean language course at the time of the investigation. The fact that participants were only Korean language students suggests a limitation but it also provides an opportunity to define domain-specific work very carefully. Seven classes at three different levels (elementary, intermediate, and advanced) participated in this study. Students completed a set of instruments as an extra credit activity in the classroom.

### Measures

#### Domain-General Creative Ideation

General creative ideation was assessed with the “Everyday” creativity scale from the *Runco Ideational Behavior Scale* (RIBS: Runco, Plucker, & Lim, 2001). The RIBS measure how frequently respondents have ideas in everyday life. The RIBS has demonstrated good reliability in various previous studies (Runco et al., 2001; Runco et al., 2014). The RIBS has also shown good construct validity (Runco et al., 2001), discriminate validity (Runco et al., 2001), and concurrent validity (Runco et al., 2014). The Everyday Creativity RIBS scale contains 20 Likert items (e.g., “I have ideas for rearranging the furniture at home,” “…ideas for a new route between home and school”). Responses were given on a 5-point Likert scale: never (1), once a year (2), once a month (3), several times a week (4), and daily (5). Internal consistency for this sample was α = .85.

A point to emphasize is that the RIBS only asks about ideas. The emphasis on ideas is explicit in each item of the RIBS (i.e., “how often do you have ideas about...”). This is an important point because it clearly distinguishes the RIBS from the Performance and Activity measures described below.

#### Domain-General Creative Performance and Activity

General creative performance and activity was assessed with the Everyday Activity scale of the *Creative Activities and Accomplishment Checklist* (CAAC, Okuda, Runco, & Berger, 1991). This checklist measures how many times students were involved in creative activities in their everyday lives (e.g., “How many times have you cooked an original dish?”). Previous findings (Okuda et al., 1991; Runco, Noble, & Luptak, 1990) indicate that the CAAC is reliable and a useful criterion of real world creative performances. It has a long history (Holland, 1961; Wallach & Wing, 1969). The Everyday scale contains 20 items, each with a 4-point Likert scale: never (1), once or twice (2), three to five times (3), and six or more times (4). Internal consistency was found to be α = .85 in this sample. Note that the Likert responses are different from those used for the RIBS. This implies that any shared variance may not reflect reliance on a common methodology. Also keep in mind what was said about the RIBS: it is focused on ideas rather than products, activities, and accomplishments.

#### Domain-Specific Creative Ideation

This was assessed within one domain. There are all kinds of domains that could be examined (e.g., math, verbal, spatial, bodily), but this particular research project was focused on the language domain, and in particular the foreign language domain. Creative ideation within this domain was assessed using a scale adapted from the original RIBS (Runco et al., 2001). For the Korean Language scale of the RIBS, 10 items from the original were selected and modified such that they targeted the foreign language domain (e.g., “I have ideas about how to apply the Korean language rules (e.g., vocabulary, grammar, social linguistic expressions) in new ways,” “…ideas about new methods for learning or teaching Korean language”). The RIBS for Korean Language was on a 5-point Likert scale: never (1), once a year (2), once a month (3), several times a week (4), and daily (5). Internal consistency was found to be α = .87 in this sample.

#### Domain-Specific Creative Performance and Activity

Creative performance and activity in the foreign language domain was assessed using a scale that was adapted from the CAAC (Okuda et al., 1991). Ten items were selected from the original CAAC (Okuda et al., 1991) and modified for the foreign language domain. The previous versions of the CAAC asked “how many times have you participated in a club or organization,” and this version modified questions so the focus was clear on the language domain (e.g., “how many times have you participated in a foreign language club or organization?”). Ratings were on a 4-point Likert scale with these options: never (1), once or twice (2), three to five times (3), and six or more times (4). Internal consistency in this study was α = .64.

#### Domain-Specific Knowledge

Here again, it was necessary to be realistic and chose an available index to estimate knowledge. There are a multitude of options, but the decision was made to obtain data from student records as estimates rather than extend the time necessary for data collection by adding an additional measure. Thus course grades in Korean classes were collected and used as the estimate of domain-specific knowledge in foreign language. The raw scores (on a scale of 1-100) were obtained from instructors, and the scores were converted to standard T-scores (*M* = 50, *SD* = 9.78).

#### Motivation

Motivation in foreign language was measured using the Korean Motivation Questionnaire (KMQ). This was modified from the Science Motivation Questionnaire II (SMQ; Glynn, Brickman, Armstrong, & Taasoobshirazi, 2011). The SMQ II includes 25 items and five motivational constructs to learn a science (i.e., intrinsic motivation, self-determination, self-efficacy, career motivation, and grade motivation). The SMQ II is reliable and valid (Glynn et al., 2011). The KMQ has the same 25 items and the five constructs as the SMQ II: intrinsic motivation (e.g., I enjoy learning Korean), self-determination (e.g., I use strategies to learn Korean well), self-efficacy (e.g., I am confident I will do well on Korean projects), career motivation (e.g., Understanding Korean will benefit me in my career), and grade motivation (e.g., Getting a good Korean grade is important to me). This assessment was on a 5-point Likert scale with these options: never (1), rarely (2), sometimes (3), usually (4), and always (5). Internal consistency was α = .88 in this sample.

#### Creative Personality

Creative personality was measured using the *Creative Personality Scale* (CPS, Gough, 1979). The CPS is reliable and has various forms of validity (Gough, 1979). Previous research has reported reliability between .73 and .81. Significant correlations have been reported between the CPS and other creativity measures (e.g., Domino’s scale for creativity, Schaefer’s scale for creativity, and Welsh’s four creativity scales). The CPS is a 30-item self-report scale on creative personality traits. It includes 18 positive for creativity traits (e.g., insightful, inventive, and humorous) and 12 negative for creativity traits (e.g., commonplace, conservative, and submissive). Participants were asked to check all traits that described themselves. They received 1 point for each positive trait and -1 point for each negative trait. Total scores ranged from -12 to 18. Internal consistency was found to be α = .76 in this sample.

## Results

### Descriptive Statistics and Correlations

As can be seen in Table 1, domain-specific knowledge was significantly and negatively correlated with General Creative Performance and Activity (*r* = −.27, *p* < .01). Motivation was significantly correlated with General Creative Ideation (*r* = .35, *p* < .01), Domain-Specific Creative Ideation (*r* = .37, *p* < .01), and Domain-Specific Creative Performance and Activity (*r* = .22, *p* < .05). Motivation also significantly correlated with CPS creative personality scores (*r* = .20, *p* < .05). Finally, creative personality significantly correlated with General Creative Ideation (*r* = .25, *p* < .01) and General Creative Performance and Activity (*r* = .24, *p* < .05).

**Table 1:** Correlation Matrix, Means, Standard Deviations, Skewness, and Kurtosis

Variable	1	2	3	4	5	6	7
1. Knowledge	__						
2. Motivation	.14	__					
3. Personality	.06	.20*	__				
4. Ideation G	−.09	.35**	.25**	__			
5. Activity G	−.27**	.02	.24*	.51**	__		
6. Ideation S	.12	.37**	.03	.32**	.12	__	
7. Activity S	−.10	.22*	−.04	.32**	.34**	.19*	__
*M*	50.00	71.40	3.09	62.16	45.71	32.09	14.95
*SD*	9.78	11.24	3.62	10.60	10.52	7.27	3.54
Skewness	−1.13	−0.38	0.20	−0.31	0.04	−0.14	0.72
Kurtosis	1.39	0.43	−0.51	−0.25	−0.31	−0.18	0.08

*Note.* Ideation G = General Creative Ideation; Activity G = General Creative Performance and Activity; Ideation S = Domain-Specific Ideation; Activity S = Domain-Specific Performance and Activity.

*p < .05. **p < .01.

The four measures of creativity (General and Domain-Specific, Ideation and Activity) were related to one another, with the exception of General Creative Performance and Activity and Domain-Specific Ideation. The correlation between General Creative Ideation and General Performance and Activity was significant (*r* = .51, *p* < .01). General Creative Ideation was also significantly correlated with Domain-Specific Creative Ideation (*r* = .32, *p* < .01) and Domain-Specific Creative Performance and Activity (*r* = .32, *p* < .01). General Creative Performance and Activity was significantly correlated with Domain-Specific Creative Performance and Activity (*r* = .34, *p* < .01). However, the relationship between General Creative Performance and Activity and Domain-Specific Creative Ideation was not significant (*r* = .12, *p* > .05). Finally, Domain-Specific Creative Ideation correlated with Domain-Specific Creative Performance and Activity (*r* = .19, *p* < .05).

### Multiple Regressions

The data were then analyzed using four multiple regression analyses. Each used one of the creativity measures as a criterion. The four models tested included domain-specific knowledge, motivation, and creative personality measures as predictors. Results are presented in Table 2.The overarching question of this investigation concerned the possibility that General Creativity differs from Domain-Specific Creativity in terms of relationships with the knowledge, motivational, and personality predictors. Most important, then, were the results from the full models. These included knowledge, motivation, and personality and explained 16% of the variance in the General Creative Ideation measure (*R*^2^ = .16, adjusted *R*^2^ = .14, *R* = .40, *p* < .01), 15% of General Creative Performance and Activity (*R*^2^ = .15, adjusted *R*^2^ = .12, *R* = .39, *p* < .01), 15% of Domain-Specific Creative Ideation (*R*^2^ = .15, adjusted *R*^2^ = .12, *R* = .38, *p* < .01), and 9% of Domain-Specific Creative Performance and Activity (*R*^2^ = .09, adjusted *R*^2^ = .06, *R* = .30, *p* < .05).

**Table 2:** Multiple Regression of General or Domain-Specific Creative Ideation or Creative Activity

DV	Predictor	B	SE	β	*t*	*R*^2^	adjusted *R*^2^	*R*
Ideation G						.16	.14	.40**
	Knowledge	−.12	.10	−.11	−1.25
	Motivation	.25	.09	.26	2.76**
	Personality	.77	.27	.26	2.86**
Activity G						.15	.12	.39**
	Knowledge	−.26	.11	−.24	−2.45*
	Motivation	−.09	.10	−.09	−0.94
	Personality	.87	.29	.30	2.97**
Ideation S						.15	.12	.38**
	Knowledge	.06	.06	.09	1.01
	Motivation	.24	.06	.36	4.04**
	Personality	−.06	.18	−.03	−0.35
Activity S						.09	.06	.30*
	Knowledge	−.05	.03	−.13	−1.34
	Motivation	.09	.03	.29	3.09**
	Personality	−.05	.09	−.05	−0.56

*Note*. Ideation G = General Creative Ideation; Activity G = General Creative Performance and Activity; Ideation S = Domain-Specific Ideation; Activity S = Domain-Specific Performance and Activity.

*p < .05. **p < .01.

Table 2 shows that motivation (β = .26, *p* < .01) and creative personality (β = .26, *p* < .01) significantly predicted General Creative Ideation, and creative personality predicted General Creative Performance and Activity (β = .30, *p* < .01). Domain-specific knowledge was negatively associated with General Creative Performance and Activity (β = −.24, *p* < .05). Motivation also predicted Domain-Specific Creative Ideation (β = .36, *p* < .01) and Domain-Specific Creative Performance and Activity (β = .29, *p* < .01).

### Canonical Correlation

A canonical correlation using domain-specific knowledge, motivation, and creative personality as predictors and the four creativity measures as criteria was significant (*Rc* = .49, *p* < .01). Thus, and quite importantly, 24.3% of variance in creativity criteria can be explained by domain knowledge, motivation and creative personality.

## Discussion

The results indicate, first, that the four measures of creativity (two representing General Creativity and two representing Domain-Specific Creativity) were largely inter-related. The exception was Domain-Specific Creative Ideation, which was not significantly correlated with General Creative Performance and Activity. The significant correlations certainly do not imply that the various measures are redundant with one another nor are interchangeable. Correlations were only moderate, which makes perfect sense. It would be a surprise if General Creative Ideation was completely independent of Domain-Specific Creative Ideation, or if General Creative Performance and Activity were not at all associated with Domain-Specific Creative Performance and Activity. It makes sense that Domain-Specific Creative Activity is associated with General Creative Ideation and Activity—after all, they are all about creativity, and general components of creativity could contribute to domain specific creativity, even if they are not redundant. Recall also Amabile’s (1990) theory that recognized both general and domain specific contributions to creative performances.

The variance explained in each of the four criterion measures (i.e., General Creative Ideation and Activity, and Domain-Specific Ideation and Activity), when the knowledge, motivation, and personality predictors were regressed, did not differ in any dramatic way (9% < *R*^2^ < 16%), but the contributions of the particular predictors did vary from criterion to criterion. Personality contributed to both General Creative Ideation and General Creative Performance and Activity, for instance, an outcome that replicates previous research (e.g., McCrae, 1987). Personality was not associated with Domain-Specific Creative Ideation nor with Domain-Specific Creative Performance and Activity, a result that is consistent with Feist’s (1998) conclusion that personality contributes to different domains in different ways. It appears that the role of personality in domain-specific creativity is not be as critical as it is in general creativity.

Domain-specific knowledge (course grade) was negatively related to General Creative Performance and Activity. This finding is in line with the previous research (e.g., Holland, 1961; Holland & Richards, 1965) showing a negative or non-significant relationship between academic achievement and general creativity (Runco & Albert, 1986). Motivation, on the other hand, was a fairly broad predictor, being related to both Domain-Specific Creative Ideation and Domain-Specific Creative Performance and Activity as well as General Creative Ideation. This too was not surprising, given findings from previous research (e.g., Rostan, 2010). Domain-specific knowledge, on the other hand, was not related to either Domain-Specific Creative Ideation or Domain-Specific Creative Performance and Activity. This was unexpected, given previous research (e.g., An et al., 2016; Vincent et al., 2002; Weisberg, 2006) on the role of domain-specific knowledge for domain-specific creativity. The difference between the present results and those of earlier investigations of domain-specific knowledge may reflect the fact that the focus here was on the number of creative activities that students voluntarily engaged in, not the quality of creative activities. Runco (1987; Runco et al., in press) previously demonstrated that the quality of creative activity does not depend on the quantity or frequency of creative activity.

Previous research compared general and domain-specific creativity by examining correlations among the measures of creativity. Support for general creativity was inferred from significant correlations among creativity measures in diverse domains (e.g., Hocevar, 1976) and from a significant relationship between general (divergent thinking test) and specific creativity measures (e.g., Plucker, 1999). Support for domain-specific creativity was inferred from non-significant correlations among creativity measures in diverse domains (e.g., Baer, 1991, 1993) and a non-significant relationship between general (divergent thinking test) and specific creativity measures (e.g., Diakidoy & Spanoudis, 2002). The logic used in those previous investigations, if applied to the present results, supports the view of general creativity. This follows from the significant correlations found between general and domain-specific ideation and activity. Yet the other approach to testing general and domain-specific creativity relies on indices of knowledge, motivation, and personality. From this angle the present results may be interpreted as supporting the domain-specific view, at least when the domain represents a foreign language. The present results were especially clear for knowledge and personality, and admittedly not as clear for motivation, but there does seem to be a need to consider the underlying indices of creativity as well as the correlations among creativity measures to test the general-specific positions.

The focus on foreign language represents a notable limitation in the present investigation. This was essentially a test of only one domain (language), and one culture for that matter. Other domains and samples would need to be tested in future research. As a measure of domain knowledge, we used students’ course grades. Although we used standardized scores for the grades, another limitation is that grading can vary from course to course, and in fact the assessment criteria may vary as well. This investigation also relied on particular measures of domain-specific knowledge, motivation, and personality as predictors of creativity. Measures that cover a wider variety of specific personality traits (e.g., HEXACO or Big 5 surveys) might uncover relationships that differ from the present results. It would be most beneficial to include assessments other than self-reports in future research. Certainly future research is needed to examine both general and specific indices to better understand the multidimensional nature of creativity.
